# Identifying characteristics associated with performing recommended practices in maternal and newborn care among health facilities in Rwanda: a cross-sectional study

**DOI:** 10.1186/1478-4491-10-13

**Published:** 2012-07-09

**Authors:** Heather L Sipsma, Leslie A Curry, Jean-Baptiste Kakoma, Erika L Linnander, Elizabeth H Bradley

**Affiliations:** 1 Department of Health Policy and Administration, School of Public Health, Yale University, New Haven, CT, USA; 2 Robert Wood Johnson Clinical Scholars Program, Department of Medicine, Yale University School of Medicine, New Haven, CT, USA; 3 School of Public Health, National University of Rwanda, Kigali, Rwanda; 4 Associate Research Scientist, 2 Church Street South, Suite 409, New Haven, CT, 06519-1717, USA

## Abstract

**Background:**

Although rates of maternal and neonatal mortality have decreased in many countries over the last two decades, they remain unacceptably high, particularly in sub-Saharan Africa. Nevertheless, we know little about the quality of facility-based maternal and newborn care in low-income countries and little about the association between quality of care and health worker training, supervision, and incentives in these settings. We therefore sought to examine the quality of facility-based maternal and newborn health care by describing the implementation of recommended practices for maternal and newborn care among health care facilities. We also aimed to determine whether increased training, supervision, and incentives for health workers were associated with implementing these recommended practices. We chose to study these aims in the Republic of Rwanda, where rates of maternal and newborn mortality are high and where substantial attention is currently focused on strengthening health workforce capacity and quality.

**Methods:**

We used data from the 2007 Rwanda Service Provision Assessment. Using observations from 455 facilities and interviews from 1357 providers, we generated descriptive statistics to describe the use of recommended practices and frequencies of provider training, supervision, and incentives in the areas of antenatal, delivery, and newborn care. We then constructed multivariable regression models to examine the associations between using recommended practices and health provider training, supervision, and incentives.

**Results:**

Use of recommended practices varied widely, and very few facilities performed all recommended practices. Furthermore, in most areas of care, less than 25% of providers reported having had any pre-service or in-service training in the last 3 years. Contrary to our hypotheses, we found no evidence that training, supervision, or incentives were consistently associated with using recommended practices.

**Conclusion:**

Our findings highlight the need to improve facility-based maternal and newborn care in Rwanda and suggest that current approaches to workforce training, supervision, and incentives may not be adequate for improving these critical practices.

## Background

Strengthening of health systems is increasingly recognized as essential for addressing the Millennium Development Goals (MDGs) [1]. Major international consortiums and initiatives, including the United Nations Global Strategy for Women’s and Children’s Health [2] and the United States of America government Global Health Initiative [3] have identified health workforce capacity building as a key element in strengthening health systems and for achieving improved health outcomes, particularly for women and children. Strategies common to programmes for enhancing health workforce capacity include workforce training, improved supervision, and the use of monetary and non-monetary incentives [4-8].

Although rates of maternal and neonatal mortality have decreased in many low- and middle-income countries over the last two decades, they remain unacceptably high, particularly in sub-Saharan Africa [9,10]. Recent evidence suggests that only 13% of countries are on track to meet MDG 5, which targets a 75% reduction in maternal mortality [11]. Furthermore, neonatal mortality remains high with almost 11,000 neonates under one month old dying per day globally [12]. Despite these alarming statistics, few studies have examined the quality of facility-based maternal and newborn care to identify specific areas of deficiency to target for intervention. Furthermore, little is known about the association between quality of care and health worker training, supervision, and incentives in low-income settings.

Accordingly, we sought to examine the quality of facility-based maternal and newborn health care by describing the use of recommended practices for maternal and newborn care among health care facilities. We also aimed to determine whether increased training, supervision, and incentives for health workers were associated with using these recommended practices for maternal and newborn care. We chose to study these aims in the Republic of Rwanda, where rates of maternal and newborn mortality are high and where substantial attention is currently focused on strengthening health workforce capacity and quality. Furthermore, in 2000, a national reproductive health policy was developed, which included training modules in prenatal care; labour and delivery; postnatal consultations; sexually transmitted infections, human immunodeficiency virus (HIV) and acquired immunodeficiency syndrome (AIDS) counselling; and prevention of mother-to-child transmission of HIV and AIDS services; as well as strengthening nursing school pre-service training and in-service training for providers, and revising reproductive health standards. Understanding the potential impact of training in addition to supervision and incentives on maternal and newborn care can inform policy and practice efforts that target human resources for health as a key lever for improving maternal and newborn health outcomes.

## Methods

### Study design and sampling

We examined facility-based maternal and newborn health care services using data from the 2007 Rwanda Service Provision Assessment (RSPA). This cross-sectional survey was designed to collect information on the performance of facilities that provided maternal, child, and reproductive services [13]. Our sample included health care facilities as well as the health service providers working at each of these facilities. Health facilities included hospitals, health centres, dispensaries, health posts, polyclinics, and clinics. Health service providers included those who directly provided client services other than laboratory tests, such as physicians and nurses. The sampling frame comprised 555 facilities, including all government-owned facilities, all private facilities that employed at least five staff members, and a random selection of one third of the private facilities that employed three or four staff members. Of the 555 facilities in the sampling frame, 538 facilities were successfully enrolled (participation rate of 97%). In each participating facility, efforts were made to interview eight providers on average, who were randomly selected from those available on the day of the interview. In facilities with fewer than eight providers, all providers were approached, yielding a total of 1935 providers from the 538 facilities (average 3.6 per facility) [13]. From this sample, we excluded facilities (and corresponding providers) that did not provide antenatal, delivery, or newborn care (n = 78). We also excluded facilities that did not have a provider interviewed who personally provided one of these types of care (n = 5 additional facilities excluded), resulting in a total sample of 455 facilities and 1357 providers.

### Data source

The 2007 RSPA data included observation data from facilities and interviews with health service providers. The observation data assessed the degree to which facility practices followed standards of care that were generally recommended for quality service delivery. A data collector observed patient visits, documented the use of procedures and examinations conducted, and discussed usual practices with staff on site. Interviews with health service providers were conducted face-to-face in the facility to assess health service provider education, experience, training, supervision, and incentives [13].

### Measures

#### Outcomes

Our outcomes included three indices to assess the use of recommended practices in antenatal care, delivery care, and newborn care. Using data derived from observations and discussions with providers, the RSPA data collector indicated with a quantitative tool whether or not specific practices were routinely performed at each of the facilities in these three areas. For each broad area (antenatal care, delivery care, and newborn care), we constructed an index based on the count of recommended practices that were routinely used at the facility. For antenatal care, eight recommended practices were assessed: measuring weight, measuring blood pressure, and testing for anaemia, syphilis, blood group, Rh factor, protein, and glucose; the resulting antenatal care index ranged from 0 (none of the practices used) to 8 (all practices used). For delivery practices, three recommended practices for the third stage of labour were assessed: administering a uterotonic drug, applying controlled cord traction, and massaging the fundus through the abdomen; this delivery care index ranged from 0 to 3. For newborn care, eight recommended practices were assessed: not suctioning with a catheter, suctioning with a bulb, weighing the newborn, not giving a full bath within 24 hours, giving no prelacteal liquids, giving the oral polio vaccine prior to discharge, giving Bacillus Calmette-Guérin prior to discharge, and offering colostrum within 1 hour of delivery; this newborn care index ranged from 0 to 8.

#### Independent variables

We assessed facility characteristics and measures of health service provider education, experience, training, supervision, and incentives. Facility characteristics included facility type (divided into hospital, health centre or polyclinic, and dispensary or health post or clinic) and facility location, which was defined by province (Northern, Southern, Eastern, Western, and Kigali City). We also used a single dichotomous variable to indicate whether the facility was government owned. We measured health service provider education and experience by self-reported number of years of primary and secondary education completed and years in current position. To assess training, health service providers also indicated whether or not they had received specific in-service or pre-service training in the last 3 years in the areas of care that they provided: antenatal care, delivery care, and/or newborn care (Table 1). To assess supervision, health service providers reported the number of times their work had been supervised in the past 6 months. This variable was categorized as 0 times, 1–2 times, 3–4 times, 5–6 times, and more than 6 times based on the response distribution and was treated as an ordinal variable for analysis based on model fit indicators. Additionally, respondents indicated whether or not the supervisor performed six specific tasks on his or her last visit; tasks included delivered supplies, checked records or reports, observed work, provided any feedback, provided updates on administrative or technical issues, and discussed problems that may have been encountered. Supervision quality was indicated by the number of tasks performed by the supervisor. To assess incentives, health service providers reported whether or not they had received any monetary and non-monetary incentives in their current position, including salary, promotion, and holidays. These two variables were treated as binary indicators in our analysis.

**Table 1:** Number and percentage of providers who report having had training in the last 3 years (n = 1357)

**Training provided in the last 3 years in:**					
**Antenatal or postpartum care (n = 1125**^**a**^**)**		**Delivery services (n = 1161**^**a**^**)**		**Newborn care (n = 906**^**a**^**)**	
Antenatal care counselling (preventive/symptomatic management)	209 (18.6%)	Care during labour and delivery	269 (23.2%)	Care of the normal newborn/neonatal care	102 (11.3%)
Antenatal care services or screening	204 (18.1%)	Use of partograph	271 (23.3%)	Neonatal resuscitation	79 (8.7%)
Complications of pregnancy	209 (18.6%)	Essential obstetric care	243 (20.9%)	Exclusive breastfeeding	88 (9.7%)
Symptom management for pregnancy	209 (18.6%)	Lifesaving skills/emergency complications	239 (20.6%)	Nutrition for the newborn of a woman infected with HIV	69 (7.6%)
Management of risk pregnancies	203 (18.0%)	Post abortion care	186 (16.0%)	Any related training	124 (13.7%)
Postpartum care	170 (15.1%)	Optimal delivery care for prevention of mother-to-child transmission of HIV or AIDS	190 (16.4%)		
Any related training	285 (25.3%)	Any related training	301 (25.9%)		

AIDS: acquired immunodeficiency syndrome; HIV: human immunodeficiency virus. ^a^The sample sizes presented include all health providers who report providing each type of care.

### Data analysis

We generated frequencies and means to describe our facility and provider samples, to examine the use of recommended practices for antenatal, delivery, and newborn care, and to describe the prevalence of health service provider training in these types of care. We then investigated the associations between our outcomes and the independent variables with bivariate analyses and multivariable regression models. Because our unit of analysis was the facility, we chose to aggregate the provider characteristics and compute means by facility. We used different modelling techniques for each outcome, based on the distributions of the measured outcome indices. The indices for antenatal care and newborn care practices followed largely normal distributions and were modelled using linear regression. The index for delivery care practices, however, followed a Poisson distribution and was therefore modelled using generalized linear modelling. Multivariable models were constructed using independent variables that we had hypothesized to be associated with care practices; we fit parsimonious models in which non-significant variables were removed one at a time, beginning with the one with the largest *P* value until all variables included in the model were significant (*P* < 0.05). We performed unweighted analyses because sampling weights were unavailable. All analyses were completed with SAS 9.2 (Carey, NC, USA).

## Results

### Sample characteristics

Of the 455 facilities included in our sample, most were health centres or polyclinics (84.0%) and were run by government authorities (63.7%). More than 90% of facilities offered antenatal or postpartum care, while 89% offered delivery care. Approximately 20% of facilities were located in each of the five regions, except with fewer facilities in Kigali City (8%) and more facilities in the Western province (27%). Almost 87% of the health service providers were nurses; 4% were physicians. On average, providers were 30 years of age with 12 years of education. Providers had been in their position for an average of 4 years.

**Figure 1:**
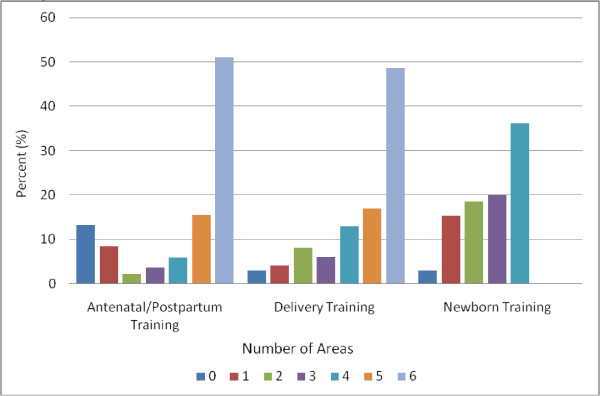
Number of areas in which training was received among providers who reported any training in each type of care.

In most areas of care, less than 25% of providers reported having had pre-service or in-service training in the last 3 years (Table 1). Training prevalence was particularly low in the area of newborn care, for which only 12% of staff who provided newborn care reported having had any training in this area in the last 3 years (Figure 1). In the last 6 months, 90% of providers reported being supervised, and almost 80% reported receiving salary supplements. Only 30% of providers reported ever having received non-monetary incentives for their work.

### Recommended practices for maternal and newborn care

**Figure 2:**
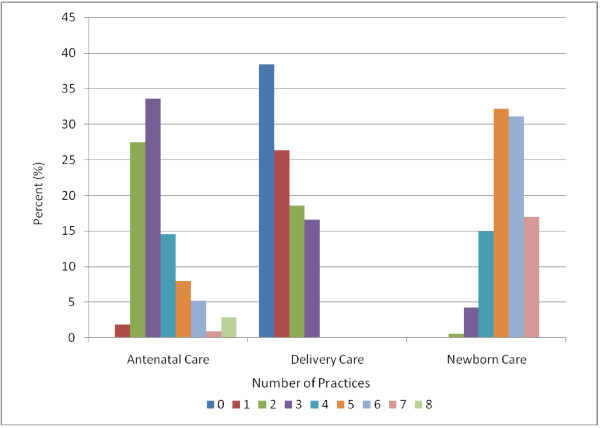
**Number of recommended practices used in facilities that delivery antenatal, delivery, and newborn care**.

Among antenatal care practices, weighing clients and taking blood pressure was either observed or reported at almost all of the facilities providing antenatal care, but routine testing was performed infrequently (Table 2). Most facilities routinely performed tests for syphilis, but fewer routinely performed tests for blood group and Rh factor. Less than 3% of facilities performed all antenatal testing practices routinely (Figure 2). Recommended procedures for the third stage of delivery were also practiced infrequently. Less than 50% of facilities routinely performed any of the recommended practices, and only 16% routinely performed all recommended practices for this stage of delivery. Newborn practices varied widely across facilities. Almost all facilities routinely weighed the newborn and had mothers offer her baby colostrum within the first hour; however, more than 95% offered prelacteal liquids to the newborn, which is not recommended as it can increase risk of early infections and interfere with exclusive breastfeeding. None of the facilities routinely performed all recommended practices for newborn care.

**Table 2:** **Prevalence of recommended practices in maternal and newborn care facilities (n = 455)**^**a**^

**Antenatal care (n = 426)**		**Delivery services (n = 404)**		**Newborn care (n = 383)**	
Weigh clients	403 (99.3%)	Administer uterotonic drug	114 (28.4%)	Give no prelacteal liquids	20 (4.2%)
Take blood pressure	399 (98.0%)	Apply controlled cord traction	158 (39.5%)	No suction by catheter	336 (88.2%)
Test blood for anaemia	91 (21.5%)	Massage fundus through	180 (44.9%)	Suction by bulb	289 (78.9%)
Test blood for syphilis	241 (56.8%)	abdomen		Weigh the newborn	374 (98.4%)
Test for blood group	23 (5.4%)	*Perform all practices*	66	Do not give full bath in first 24 h	232 (60.9%)
Test for Rh factor	19 (4.5%)		(16.6%)	Give oral polio vaccine prior to discharge	252 (66.3%)
Test urine for protein	130 (30.8%)			Give Bacillus Calmette-Guérin prior to discharge	199 (52.4%)
Test urine for glucose	68 (16.2%)			Give colostrum within 1 h	359 (94.5%)
*Perform all practices*	12 (2.8%)			*Perform all practices*	0 (0.0%)

^a^Activity either observed or reported.

### Factors associated with recommended practices

#### Provider training, supervision, and incentives

Neither provider training nor supervision was statistically associated (*P* > 0.05) with the use of recommended practices for antenatal care, delivery, or newborn care (Tables 3, 4 and 5). Non-monetary incentives were significantly associated with more recommended practices, but only among newborn care, and we found no evidence that monetary incentives were associated with using more recommended practices in any of the areas examined.

**Table 3:** Linear regression analysis: factors associated with number of antenatal care practices facilities (n = 400)

	**Mean number of antenatal care practices**	**Unadjusted coefficient (SE)**	**Adjusted coefficient (SE)**
**Facility characteristics**			
*Facility type*			
Hospital	6.00	3.26 (0.64)**	2.46 (0.62)**
Health centre	3.33	REF	REF
Dispensary	2.97	−0.37 (0.27)	−0.55 (0.27)*
*Province*			
Northern	3.21	−1.32 (0.29)**	−1.14 (0.29)**
Southern	3.76	−0.76 (0.28)**	−0.69 (0.28)*
Eastern	2.67	−1.86 (0.29)**	−1.66 (0.28)**
Western	3.26	−1.26 (0.28)**	−1.00 (0.27)**
Kigali City	4.45	REF	REF
*Managing authority*			
Government owned	3.24	−0.34 (0.15)*	
Non-government owned	3.55	REF	
**Provider education and experience**			
Mean years of education		0.17 (0.06)**	
Mean years in current position		0.07 (0.02)**	0.05 (0.02)**
**Provider training, supervision, and incentives**			
Any antenatal training		0.00 (0.26)	
Number of times supervised		−0.10 (0.08)	
Supervision quality		−0.02 (0.06)	
Ever received salary supplement		−0.23 (0.24)	
Ever received non-monetary incentive		−0.17 (0.22)	

SE, standard error; *P < 0.05; ** *P* ≤ 0.01.

**Table 4:** Poisson regression analysis: factors associated with number of delivery practices facilities (n = 398)

	**Mean number of delivery practices**	**Unadjusted rate ratio (95% CI)**	**Adjusted rate ratio (95% CI)**
**Facility characteristics**			
*Facility type*			
Hospital	1.50	1.37 (1.05, 1.79)*	
Health centre	1.10	REF	
Dispensary	1.05	0.96 (0.59, 1.54)	
*Province*			
Northern	0.74	0.74 (0.48, 1.12)	0.74 (0.48, 1.12)
Southern	0.94	0.94 (0.64, 1.39)	0.94 (0.64, 1.39)
Eastern	1.23	1.23 (0.83, 1.82)	1.23 (0.83, 1.82)
Western	1.54	1.54 (1.06, 2.25)*	1.54 (1.06, 2.25)*
Kigali City	1.00	REF	REF
*Managing authority*			
Government owned	1.10	0.92 (0.76, 1.11)	
Non-government owned	1.20	REF	
**Provider education and experience**			
Mean years of education		1.02 (0.93, 1.12)	
Mean years in current position		0.98 (0.95, 1.02)	
**Provider training, supervision, and incentives**			
Any training in delivery care		1.21 (0.88, 1.68)	
Number of times supervised		0.87 (0.80, 0.98)*	
Supervision quality		1.00 (0.92, 1.08)	
Ever received salary supplement		0.95 (0.68, 1.32)	
Ever received non-monetary incentive		0.81 (0.59, 1.10)	

CI, confidence interval; *P < 0.05; **P ≤ 0.01.

**Table 5:** Linear regression analysis: factors associated with newborn care practices facilities (n = 376)

	**Mean number of newborn care practices**	**Unadjusted coefficient (SE)**	**Adjusted coefficient (SE)**
**Facility characteristics**			
*Facility type*			
Hospital	4.76	−0.77 (0.19)**	−0.78 (0.19)**
Health centre	5.52	REF	REF
Dispensary	4.27	−1.25 (0.28)**	−1.34 (0.28)**
*Province*			
Northern	5.46	0.00 (0.26)	
Southern	5.84	0.39 (0.25)	
Eastern	5.41	−0.02 (0.25)	
Western	4.92	−0.55 (0.25)*	
Kigali City	5.45	REF	
*Managing authority*			
Government owned	5.51	0.32 (0.12)**	
Non-government owned	5.20	REF	
**Provider education and experience**			
Mean years of education		0.02 (0.04)	
Mean years in current position		0.00 (0.02)	
**Provider training, supervision, and incentives**			
Any training in newborn care		−0.34 (0.20)	
Number of times supervised		0.18 (0.06)**	
Supervision quality		0.06 (0.04)	
Ever received salary supplement		0.13 (0.18)	
Ever received non-monetary incentive		0.41 (0.16)*	0.52 (0.15)**

SE , standard error; *P < 0.05; **P < 0.01.

#### Provider education and experience

Provider education was not statistically associated with the use of recommended practices in any area. In facilities with providers with more years of experience on average, significantly more of the recommended antenatal care practices were performed, although this trend was not apparent for delivery or newborn care practices.

#### Facility characteristics

Facility characteristics were significantly associated with performing recommended practices. Hospital facilities performed more of the antenatal care practices on average than health centres, and dispensaries performed fewer of these practices than health centres (Table 3). Delivery care practices did not vary significantly by facility type (Table 4), but hospitals and dispensaries performed fewer newborn care practices than health centres (Table 5). Facility location was inconsistently associated with recommended practice performance. For instance, facilities in Kigali City performed more routine practices in antenatal care but fewer practices in delivery care compared with the Western province. Government-owned facilities did not differ significantly from non-government facilities for any type of care.

## Discussion

Our results indicate that many practices recommended for high quality maternal and newborn care were not routinely performed in Rwandan health facilities, and fewer than 17% of facilities performed all recommended practices in antenatal care, delivery, or newborn care. The limited use of recommended practices was apparent in both government-owned and non-governmental facilities and across all provinces of Rwanda. Furthermore, although the rural provinces (Northern, Southern, Eastern, and Western) performed fewer antenatal care practices than the urban area of Kigali City, this association was not consistent for delivery or newborn care practices. The better performance of antenatal care in Kigali City may be attributable to the relatively recent (since 1997) training of midwives, the majority of whom live and practice in Kigali City. Given the importance of high quality facility-based care for achieving the MDGs in the areas of maternal and child health, finding ways to promote more consistent use of recommended clinical practices is paramount.

Despite the emphasis in current health policy and practice on enhancing workforce performance through training, we found no evidence that training was statistically associated with the increased use of recommended practices. This lack of association, however, is consistent with a small body of literature [14-16] and suggests the need to better understand effective training mechanisms. Furthermore, supervision and monetary incentives were also not associated with greater use of recommended practices. Although non-monetary incentives were associated with increased use in newborn care, this effect was not consistent across all areas. These results were surprising and not consistent with our a priori hypotheses.

What could explain the lack of associations found between our hypothesized exposures and outcomes? First, it is possible that the impact of training occurs only after a critical mass of staff is trained. Perhaps the needed critical mass had not been achieved, limiting the potential impact of those trained on overall facility-level practices. Second, we did not examine the quality of the training or incentive systems, only their presence or absence, and examined only limited measures of supervision quality. If the training was overly theoretical and classroom-based, its impact on practices may have been more limited. Similarly, if the supervision was inconsistent or incentives were too small, their influence might be modest. Last, because health providers work within a larger system, their training, supervision, and incentives may be inadequate for creating needed changes at the facility level. Additional factors such as effective management and an organizational culture of quality improvement may also be necessary to promote adherence to recommended practices. As bilateral and multilateral agencies focus on capacity building, it is critical that provider training be designed and implemented with attention to systems-level factors so that providers’ new skills and knowledge may be effectively translated into practice.

Our findings should be interpreted in light of several limitations. First, the providers interviewed may not have been representative of the staff who typically provided health services at each facility, although the RSPA made every effort to collect an unbiased sample of provider data. Second, we did not adjust for the availability of equipment and supplies, which may have influenced the use of recommended practices, even though we focused on practices that required minimal equipment and supplies. Third, we examined training in the last 3 years, which we viewed as more central to current practices; however, this may have provided an inaccurate view for workers who were trained more than 3 years ago. Nevertheless, among health workers who had more than 3 years of experience, we conducted the same analysis and results were largely unchanged. Also, because the average time in the position was 4 years, the measure of training in the last 3 years seemed most relevant for our research. Fourth, our measures on training, supervision, and incentives may have been inadequate as we were limited to the data available in the RSPA. These measures primarily indicated only their presence or absence and not the quality of their design or implementation; however, to the best of our knowledge, these indicators are the best measures available at a national level. Additionally, the measurement of incentives could have captured incentives that were related to actions other than the measured practices and therefore would not be expected to be associated with these practices. Incentives designed specifically for the measured practices may have had other results; however, we were limited to the data available in the RSPA, the most contemporary national dataset on this topic in Rwanda. Future research would benefit from more extensive quantitative and qualitative measures to better understand these associations. Furthermore, based on the cross-sectional nature of the data, we are unable to assert causality. For example, it is possible that the worst performing facilities were the ones that implemented training or incentives in an effort to improve, which could in part explain our null findings. Future work would benefit from prospective research that would allow controlling for baseline conditions. Finally, it is possible that these data underestimate current practices, as they reflect conditions in 2007 and Rwanda has been recovering rapidly from the genocide a decade earlier. Nevertheless, the data are the most recent national data available.

## Conclusions

Our results indicate that many practices recommended for high quality maternal and newborn care were not routinely performed in Rwandan health facilities. They also add to our understanding that health worker training, supervision, and incentives are complex interventions, and it is difficult to measure their effect or the reason for a lack of effect on quality of care. Strategies for effectively translating these guidelines into practice are critical and we suggest the importance of implementing better tracking tools, such as real-time checklists, to improve quality of care. Future work should also study high performing facilities to identify characteristics that may suggest reasons for them performing comparatively well. Establishing and monitoring facility-level quality of care, including health providers’ use of recommended clinical practices, will be of critical importance as movement toward achieving the MDGs will require marked improvements in the quality of maternal and newborn care.

## Competing interests

The authors declare that they have no competing interests.

## Authors’ contributions

HS and EB conceptualized and designed the data. HS acquired the data and performed the statistical analysis. HS and EB drafted the manuscript. All authors substantively revised the manuscript and approve of its final form. All authors read and approved the final manuscript.
